# Hypertension Management in the High Cardiovascular Risk Population

**DOI:** 10.1155/2013/382802

**Published:** 2013-02-06

**Authors:** Ilir Maraj, John N. Makaryus, Anthony Ashkar, Samy I. McFarlane, Amgad N. Makaryus

**Affiliations:** ^1^Division of Endocrinology, Diabetes, and Hypertension, SUNY Downstate Medical Center, Brooklyn, NY 11203, USA; ^2^Department of Cardiology, North Shore University Hospital, Manhasset, NY 11030, USA; ^3^Hofstra North Shore-LIJ School of Medicine and the Department of Cardiology, Nassau University Medical Center, 2201 Hempstead Turnpike, East Meadow, NY 11554, USA

## Abstract

The incidence of hypertension is increasing every year. Blood pressure (BP) control is an important therapeutic goal for the slowing of progression as well as for the prevention of Cardiovascular disease. The management of hypertension in the high cardiovascular risk population remains a real challenge as the population continues to age, the incidence of diabetes increases, and more and more people survive acute myocardial infarction. We will review hypertension management in the high cardiovascular risk population: patients with coronary heart disease (CHD) and heart failure (HF) as well as in diabetic patients.

## 1. Introduction

Over 65 million adult Americans or approximately one-fourth of the US population has hypertension. The impact of hypertension in cardiovascular morbidity and mortality is higher than any other cardiovascular risk factor including traditional factors such as obesity and dyslipidemia (Table 1) and nontraditional risk factors such as increased inflammation and hypercoagulable states (Table 2). Among individuals aged 40–90 years, each 20/10 mmHg rise in blood pressure (BP) doubles the risk of fatal coronary events [1]. Hypertension has been shown to cause decreased vascular compliance and endothelial injury [1, 2].

Endothelial injury is one of the main mechanisms in the pathogenesis of atherosclerosis and coronary heart disease (CHD). Endothelial injury impairs synthesis of the potent vasodilator nitric oxide. This in turn leads to further inflammation and thrombosis by the way of reactive oxygen species and multiple inflammatory markers [3]. Therefore, endothelial injury caused by hypertension (HTN) leads to a cascade of events forming the foundation of CHD development [3].

Another mechanism in the development of CHD is the renin-angiotensin aldosterone system (RAAS). Studies have shown that angiotensin II increases BP and also generates reactive oxygen species which contribute to opposing the beneficial vascular effects of nitric oxide. Angiotensin II has been shown to increase arterial wall stiffness, thus impairing vascular compliance. In addition, angiotensin II contributes to the development of insulin resistance and stimulates production of proinflammatory molecules that cause vascular inflammation and coagulopathy [2, 3]. The treatment and management of these patients focus on targeting and ameliorating of these pathologic mechanisms in each of these three main high cardiovascular risk populations: (1) patients with CHD, (2) patients with HF, and (3) diabetic patients.

## 2. Therapeutic Interventions

Nonpharmacologic interventions are recommended as primary and adjunctive treatment options for successfully lowering blood pressure in all hypertensive patients. These interventions include weight reduction, increase in physical activity, and restriction of sodium, following the DASH (Dietary Approaches to Stop Hypertension) diet, tobacco cessation, and reduction of alcohol intake [4].

Exercise improves cardiac function and reduces blood pressure and cardiac overload by a variety of mechanisms, including reducing arterial stiffness. Although the mechanism is not entirely clear, evidence suggests that exercise improves coronary artery flow reserve in CHD patients [5, 6].

Pharmacological treatment is inevitable in high-risk populations such as those with CHD. The recommended target blood pressure for individuals with CHD or CHD equivalents is <130/80 mmHg. The remainder of this paper will focus on the pharmacologic treatment in each of these high risk populations: (1) patients with CHD, (2) patients with HF, and (3) diabetic patients.

## 3. Management of Hypertension in Patients with CHD

Both the Seventh Report of the Joint National Committee on Prevention, Detection, Evaluation and Treatment of High Blood Pressure (JNC 7) and the American Heart Association (AHA) guidelines stress the importance of antihypertensive therapy in the high-risk population, including coronary heart disease. The AHA recommended a target BP of 130/80 mmHg in patients with CHD as for the other high risk populations [7, 8].

The goals for treatment of hypertension in patients with CHD are to lower blood pressure, reduce ischemia, and prevent cardiovascular events and death. To achieve these goals, both nonpharmacological interventions and pharmacologic therapy are recommended.

### 3.1. Beta-Blockers

The first-line therapy in the treatment of hypertension in patients with CHD should be beta-blockers unless contraindicated. Beta-blockers comprise a heterogenous class of antihypertensive agents that decrease heart rate, reduce myocardial oxygen consumption, and increase the diastolic filling period, thus enhancing the coronary flow. The cardioselective beta-blockers without intrinsic sympathomimetic activity are generally preferred. Beta-blockers have been shown to improve survival, decrease the risk of recurrent myocardial infarction (MI), and decrease the incidence of sudden cardiac death among patients with CHD [9–12]. However, in patients with no CHD, there is no sufficient evidence for the cardioprotective effect of beta-blockers. While metoprolol, carvedilol, and bisoprolol are shown to improve outcomes in HF patients, results of the ASCOTBPLA (Anglo-Scandinavian Cardiac Outcomes Trial—Blood Pressure-Lowering Arm) suggest that atenolol may be marginally inferior to amlodipine in reducing cardiovascular events [13, 14].

### 3.2. Angiotensin-Converting Enzyme Inhibitors

ACE inhibitors are recommended for all patients after MI. Three major trials have addressed the use of ACE inhibitors for hypertension in patients with or at high risk of CHD. In the HOPE trial (Heart Outcome Prevention Evaluation) that involved 9297 patients 55 years of age or older, who had evidence of vascular disease or DM plus an additional cardiovascular risk factor, treatment with ramipril was associated with a 22% reduction in the composite endpoint of cardiovascular death, MI, and stroke (*P* < 0.001). Ramipril therapy was associated with small (3/2 mmHg) reduction in blood pressure but a significant reduction in cardiovascular (CVS) death, stroke, and MI [15].

The EUROPA trial (European Trial on Reduction of Cardiac events with Perindopril in Stable Coronary Artery Disease) investigators randomized 12,218 patients to perindopril or placebo. Mean followup was 4.2 years. Perindopril therapy was associated with a 20% relative risk reduction in the primary endpoint, a composite of cardiovascular death, MI, or cardiac arrest (*P* = 0.003). The benefit of treatment with perindopril was comparable for patients with or without hypertension [16].

The PEACE trial (Prevention of Events with Angiotensin-Converting Enzyme Inhibition) involved patients who had stable CHD and normal or slightly reduced LV function and randomized these patients to trandolapril or placebo. Median followup was 4.8 years. There was a 4.4/3.6 mmHg mean reduction in BP noted in patients receiving trandolapril; however, there was no difference between trandolapril and placebo in the incidence of cardiovascular events. This study concluded that ACE inhibitors might not be necessary in low-risk CHD patients with normal LV function. Thus ACE inhibitors are indicated in all hypertensive patients with acute MI, especially in those patients with depressed LV systolic dysfunction (LVEF < 40%) [17].

### 3.3. Angiotensin II Receptor Blockers

The angiotensin receptor II blockers (ARBs) have been shown to be an effective treatment for hypertension in patients with CHD and HF in patients who are intolerant to ACE inhibitors [18, 19]. The VALUE trial (Valsartan Antihypertensive Long-Term Use Evaluation) showed that there was no difference in cardiac mortality and morbidity in patients with hypertension and high risk of cardiovascular events who were treated with valsartan versus amlodipine, eventhough the BP-lowering effect of amlodipine was greater than that of valsartan [19, 20].

The VALIANT trial (Valsartan in Acute Myocardial Infarction) showed that valsartan and captopril are equally effective in reducing the rates of death and cardiovascular events in patients with high risk after MI. However, the VALIANT trial also revealed that when valsartan and captopril were given in combination, they did not improve survival, but increased the rate of adverse events in these patients [21].

The ONTARGET trial (Ongoing Telmisartan Alone and in Combination with Ramipril Global Endpoint Trial) established the role of ARBs in reducing cardiovascular events in high-risk patients. This trial showed that telmisartan is comparable with ramipril in patients with cardiovascular disease or diabetes, with fewer cough and angioedema events. The combination of two drugs did not improve the outcomes and was associated with more adverse events [22].

The TRANSCEND trial (Telmisartan Randomized Assessment Study in ACE Intolerant Subjects with Cardiovascular Disease) validates the role of ARBs in reducing the cardiovascular risk in high-risk patients. It demonstrated that treatment with telmisartan in patients receiving the standard of care resulted in 8% reduction in the composite endpoint of cardiovascular death, myocardial infarction, stroke, and hospitalization for congestive heart failure. This reduction, however, was not statistically significant (*P* = 0.216; HR 0.92) compared to patients receiving placebo in addition to the standard of care [23].

### 3.4. Calcium Channel Blockers

The dihydropyridine calcium channel blockers (CCBs), amlodipine and nifedipine, can be added to the beta-blocker regimen, if blood pressure remains uncontrolled in patients with CHD. The nondihydropyridine agents such as diltiazem and verapamil can be substituted for beta-blockers when side effects develop or there are contraindications. CCBs reduce blood pressure by causing vasodilation and decreasing peripheral vascular resistance, thus reducing the myocardial oxygen demand [24].

According to the ALLHAT trial, in the primary prevention of cardiovascular events, amlodipine was equivalent to thiazide diuretics and ACE inhibitors [25]. Primary prevention with verapamil-based therapy was shown to be similar to diuretic or beta-blocker-based therapy.

The CONVINCE (Controlled-Onset Verapamil in Cardiovascular Endpoint), NORDIL (the Nordic diltiazem study), and INVEST (International Verapamil SR/Trandolapril Study) trials showed that, in CCB-basedtherapy, outcomes were not different from beta-blockers. CCBs are the alternatives to beta-blockers in the treatment of HTN in patients with CHD but are not recommended for secondary cardiac prevention because of their inability to prevent ventricular dilatation, especially when compared to ACE inhibitors or ARBs. CCBs should be avoided in patients with acute MI, pulmonary edema, and LV dysfunction [26–28].

### 3.5. Diuretics

The effectiveness of thiazide diuretics in controlling blood pressure and preventing cardiovascular events has been demonstrated in several studies.

The ALLHAT (the Antihypertensive and Lipid-Lowering Treatment to Prevent Heart Attack) trial demonstrated that there were no significant differences among the thiazide diuretic chlortalidone, the CCB amlodipine, and the ACE inhibitor lisinopril in the combined outcomes of fatal CHD and nonfatal MI [16].

### 3.6. Nitrates

Nitrates are the preferred choice for the management of acute hypertension, acute relief of angina, or chronic angina that cannot be controlled with beta-blockers and calcium channel blockers. The GISSI-3 trial (Gruppo Italiano per lo Studio della Sopravvivenza nell'Infarcto Miocardico) and the ISIS-4 trial (International Study of Infarct Survival) found no difference in mortality in patients treated with nitrates versus placebo [29]. Therefore the American College of Cardiology/American Heart Association (ACC/AHA) guidelines recommend that nitrates should not be used alone for the management of hypertension in CHD patients at the expense of agents with proven benefits on outcomes [29].

## 4. Management of Hypertension in HF Patients

The goal of therapy in hypertensive patients with heart failure and systolic dysfunction is to decrease preload and afterload. Therapy for hypertension in HF patients includes beta-blockers, ACE inhibitors, ARBs, diuretics, hydralazine, and isosorbide dinitrate.

### 4.1. Beta-Blockers

Beta-blockers are considered the standard therapy in managing hypertensive patients with heart failure, who have no contraindications to the use of these agents and who are not in acute decompensated HF. Most commonly used beta-blockers in patients with heart failure include bisoprolol, metoprolol, and carvedilol. Bisoprolol and metoprolol are beta1-selective blockers without significant intrinsic sympathetic activity or vasodilating properties. Carvedilol has antagonist activity against alpha1, beta1, and beta2 receptors, as well as some antioxidant activity.

A number of large randomized trials showed a mortality benefit in using beta-blockers in patients with heart failure. The Cardiac Insufficiency Bisoprolol Study (CIBIS) was the first true mortality trial of beta-blockers. The CIBIS demonstrated the safety of bisoprolol, along with a significant improvement in the New York Heart Association (NYHA) class and a decrease in hospitalization. The CIBIS-II trial validated CIBIS. It involved approximately four times as many patients as CIBIS, included more patients with NYHA class IV symptoms, and used a higher dosage of bisoprolol. The improvement in mortality was statistically significant [30].

The Metoprolol in Dilated Cardiomyopathy (MDC) and the Metoprolol CR/XL Randomised Intervention Trial in Congestive Heart Failure (MERIT-HF) showed the impact of metoprolol on mortality in patients with heart failure. MERIT-HF was a larger trial, which used metoprolol succinate and was stopped prematurely as the absolute mortality rate was 7.2% per patient-year of followup in the treatment group compared with 11% in the placebo group. This difference was statistically significant [31, 32].

In the COPERNICUS trial (Carvedilol Prospective Randomized Cumulative Survival), patients were randomized to carvedilol versus placebo. This trial was terminated early as carvedilol showed a large mortality benefit in the treatment group [33].

### 4.2. Angiotensin-Converting Enzyme Inhibitors

ACE inhibitors are one of the main therapies in managing hypertensive patients with HF. ACE inhibitors increase cardiac output, decrease congestion due to their vasodilator (venodilatation) activity, reduce the rate of progressive cardiac dysfunction, and improve cardiovascular mortality. Multiple trials have shown the benefit of ACE inhibitors in patients with HF.

The CONSENSUS trial (Cooperative North Scandinavian Enalapril Survival Study) evaluated the influence of the angiotensin-converting-enzyme inhibitor enalapril in the prognosis of severe congestive heart failure (New York Heart Association's class IV). In this trial, 253 patients were assigned to receive either placebo or enalapril. Conventional treatment for heart failure, including the use of other vasodilators, was continued in both groups. This study concluded that the addition of enalapril to the conventional therapy in patients with HF reduces mortality and improves symptoms and it was also associated with significant reductions in the NYHA class [34].

In the SOLVD (Studies of Left Ventricular Dysfunction) trial, 2569 patients with symptomatic NYHA class II to III HF with LVEF 35% were evaluated. When compared to placebo, enalapril resulted in a significant reduction in all-cause mortality (35 versus 40 percent, risk reduction 16 percent, 95% confidence interval) [35].

### 4.3. Angiotensin II Receptor Blockers

The angiotensin II receptor blockers (ARBs) appear to be as effective as ACE inhibitors for the management of HTN in patients with HF. They are mostly used in patients intolerant to ACE inhibitors.

### 4.4. Hydralazine and Isosorbide Dinitrate

The combination of hydralazine and isosorbide dinitrate prolongs survival and is found to be effective in African-American patients with heart failure already being treated with the standard therapy. The African-American Heart Failure Trial (A-HeFT) involved a total of 1050 black patients who had the New York Heart Association's class III or IV heart failure with dilated ventricles, which were randomly assigned to receive a fixed dose of isosorbide dinitrate plus hydralazine or placebo in addition to the standard therapy for heart failure. The primary endpoint was a composite score made up of weighted values for death from any cause, a first hospitalization for heart failure, and change in quality of life. The group treated with hydralazine plus isosorbide dinitrate versus placebo showed decreased mortality [36].

### 4.5. Aldosterone Antagonists

Spironolactone and eplerenone improve survival in patients with advanced HF. Two large trials have evaluated the use of an aldosterone receptor antagonist in patients with heart failure in addition to an ACE inhibitor: the Randomized Aldactone Evaluation Study (RALES) and the Eplerenone Heart Failure Efficacy and Survival Study (EPHESUS).

The RALES trial showed that a blockade of aldosterone receptors by spironolactone, in addition to the standard therapy, reduces significantly the risk of morbidity and mortality among patients with severe heart failure and a left ventricular ejection fraction of no more than 35 percent [37]. In the EPHESUS trial, the addition of eplerenone to the optimal medical therapy was shown to reduce morbidity and mortality among patients with acute myocardial infarction complicated by left ventricular dysfunction and heart failure [38].

### 4.6. Diuretics and Calcium Channel Blockers

Diuretic therapy (loop diuretics-furosemide) is used mostly for signs of fluid overload and improvement in symptoms. There is no evidence that loop diuretics improve survival in patients with HF. CCBs also showed no mortality benefit in the treatment of hypertensive patients with HF.

### 4.7. Therapy for Heart Failure Patients with Diastolic Dysfunction

While the focus of therapy for HF patients has been on systolic dysfunction, hypertensive patients with diastolic dysfunction should be managed with beta-blockers, ARBs, and verapamil. These agents have been shown to improve left ventricular compliance, regress left ventricular hypertrophy, and decrease myocardial oxygen demand [39].

## 5. Management of Hypertension in Diabetic Patients

More than 10 million adults in the US have diabetes. Hypertension is twice as common in patients with diabetes compared to the general population. Clinical trials have demonstrated that the aggressive blood-pressure-lowering target of <130/80 mmHg in diabetic patients may help prevent diabetic complications [40].

### 5.1. Angiotensin-Converting Enzyme Inhibitors/Angiotensin II Receptor Blockers

Many studies have shown clear benefit of ACE inhibitors and ARBs in reducing microvascular and macrovascular complications in hypertensive patients with type I and II diabetes. In patients with microalbuminuria or clinical nephropathy, both ACE inhibitors and ARBs are considered a first-line therapy for the prevention of nephropathy progression according to guidelines from the American Diabetes Association (ADA), the National Kidney Foundation (NKF), and JNC 7 [41]. The HOPE (The Heart Outcomes Prevention Evaluation) trial supported the above recommendations. Many trials have also shown ACE inhibitors to be of significantly greater benefit when compared with other antihypertensive agents in the reduction of acute MI, cardiovascular events, and all-cause mortality in diabetic patients.

The UKPDS (the United Kingdom Prospective Diabetes Study) trial on the other hand showed no difference in terms of reduction in microvascular and macrovascular complications when it compared captopril with atenolol [42].

The CALM (Candesartan and Lisinopril Microalbuminuria) study showed that candesartan was as effective as lisinopril in blood pressure reduction and minimization of microalbuminuria [43].

One area of concern is the use of ACE inhibitors and ARBs in patients with bilateral renal artery stenosis. To help detect the presence of undiagnosed bilateral renal artery stenosis, the serum creatinine level should be monitored at baseline and one week after the initiation of therapy with any of these agents.

The RAAS blockade also may play a role in the prevention of diabetes. In a *post hoc* analysis of the HOPE trial, ramipril was associated with a 34% reduction in the risk of new-onset diabetes when compared with placebo (RR 0.66, 95% CI: 0.51–0.85; *P* < 0.001). A meta-analysis of 13 studies involving approximately 67,000 patients showed that ACEi and ARBs significantly reduced new-onset diabetes in patients with hypertension or other cardiovascular risk factors [44, 45].

### 5.2. Diuretics

Diuretics are effective in the treatment of hypertension in patients with diabetes. They enhance the efficacy of other antihypertensives. Lower doses of thiazide diuretics are well tolerated. The ALLHAT trial showed that chlorthalidone, amlodipine, and lisinopril are equal in terms of primary outcomes of CHD, death, and nonfatal MI. In addition, chlorthalidone was found to be superior to amlodipine and lisinopril in preventing new-onset heart failure. Chlorthalidone was associated with a mild rise in plasma glucose [16]. However, thiazide diuretics are not as effective in patients with chronic kidney disease. Loop diuretics are preferred in those cases.

### 5.3. Calcium Channel Blockers

Two trials, the ABCD (Appropriate Blood Pressure Control in Diabetes) trial and the FACET (Fosinopril versus Amlodipine Cardiovascular Events Randomized trial) demonstrated no significant reduction in cardiovascular events with fosinopril when compared to amlodipine [46, 47].

In contrast, the HOT (Hypertension Optimal Treatment) trial concluded that the use of dihydropyridine CCBs as monotherapy or in combination with another agent was associated with a reduction in the cardiovascular risk in these patients [48].

### 5.4. Beta-Blockers

Beta-blockers are effective means of therapy in controlling blood pressure in diabetic patients. The UKPDS41 study showed that, in patients with DM type II, atenolol was as effective as captopril regarding blood pressure control and protection against microvascular complications. The LIFE trial demonstrated that losartan provides significantly more cardiovascular protection compared to atenolol [49].

Cardioselective beta-blockers are associated with less blunting of hypoglycemic awareness and less elevation of lipid and glucose levels. Carvedilol that is a nonselective beta-blocker and alpha-1 adrenergic antagonist may have some advantages compared to other beta-blockers when used in diabetes patients. It has been shown to cause fewer alterations in lipid and glucose levels compared with the traditional beta-blockers [50].

### 5.5. Combination Therapy

Most patients require more than one blood pressure agent to achieve adequate blood pressure control. The choice of the antihypertensive therapy in diabetic patients is based upon their ability to prevent cardiovascular events and to slow progression of nephropathy if present.

The ACCOMPLISH (Avoiding Cardiovascular Events through Combination Therapy in Patients Living with Systolic Hypertension) trial demonstrated the superiority of a combination of an ACE inhibitor with a CCB over ACE inhibitor plus a thiazide diuretic. In this trial, 11,506 patients with hypertension (60% had diabetes) were assigned to a combination therapy consisting of either benazepril plus amlodipine or benazepril plus low-dose hydrochlorothiazide. The primary endpoint was a composite death from cardiovascular causes, nonfatal MI, nonfatal stroke, hospitalization for angina, resuscitation after sudden cardiac arrest, and coronary revascularization. The trial was terminated early, after a mean followup of 36 months, as it showed overwhelming efficacy in favor of the benazepril-amlodipine combination [51].

## 6. Conclusion

The goal of blood pressure therapy in patients with CHD, HF, and DM is to improve mortality and to reduce cardiovascular events. The target blood pressure in hypertensive patients with CHD, HF, and DM is <130/80 mmHg with caution exercised in lowering diastolic blood pressure <70 mmHg. Treatment modalities should be individualized based on co-morbidities and tolerability.

The treatment regimen in patients with CHD should include beta-blockers, ACEi, ARBs, and diuretics (thiazide diuretics). CCBs should be used as an alternative or added to the basic regimen. Nitrates should be used to relieve the ischemic pain. Hypertensive patients with systolic heart failure are treated mostly with ACEi, ARBs, beta-blockers, diuretics, and aldosterone antagonists. Appropriate agents also include hydralazine and isosorbide dinitrate as a supplement to the basic therapy. The optimal therapy for hypertensive patients with diastolic dysfunction includes beta-blockers, ARBs, and verapamil. These agents have been shown to improve left ventricular compliance, regress left ventricular hypertrophy, and decrease myocardial oxygen demand.

The choice of an antihypertensive agent used in patients with diabetes mellitus depends on their ability to decrease cardiovascular events, improve mortality, and to slow progression of nephropathy. ACEi or ARBs should be the initial therapy in hypertensive patients with diabetes mellitus and microalbuminuria. If beta-blockers are to be given, carvedilol is shown to be superior compared to traditional selective beta-blockers. Also, CCBs and thiazide diuretics when used as a combination therapy with ACEi improved the cardiovascular events.

As can be seen by the review of prior trials and recommendations, appropriate management of these patients through targeted pharmacologic and nonpharmacologic therapies to improve mortality and reduce cardiovascular events is the optimal approach to treatment.

## Figures and Tables

**Table 1:** Traditional risk factors for cardiovascular disease.

(1) Age	
(2) Male gender	
(3) Postmenopausal state	
(4) Smoking	
(5) Family history of premature coronary artery disease	
(6) Hypertension	
(7) Diabetes mellitus	
(8) Insulin resistance	
(9) Central obesity	
(10) Low level of high-density lipoprotein	
(11) High triglyceride levels	
(12) Small dense low-density lipoprotein	

**Table 2:** Nontraditional risk factors for cardiovascular disease.

Endothelial dysfunction	
Microalbuminuria	
Increased Apolipoprotein B levels	
Increased fibrinogen levels	
Increased plasma activator inhibitor-1 level	
Increased C-reactive protein and other inflammatory markers	
Absence of nocturnal dipping in blood pressure and pulse	
Salt sensitivity	
Left ventricular hypertrophy	
